# Oral GLP-1-Based Therapeutics in the Obesity–Metabolic Syndrome–Diabetes Continuum: Translational Advances, Clinical Barriers, and Emerging Strategies

**DOI:** 10.3390/ph19050732

**Published:** 2026-05-07

**Authors:** Syed Arman Rabbani, Manita Saini, Mohamed El-Tanani, Rakesh Kumar, Ismail Matalka, Yahia El-Tanani, Shrestha Sharma, Manfredi Rizzo

**Affiliations:** 1 Clinical Pharmacy & Pharmacology, RAK College of Pharmacy, Ras Al Khaimah Medical and Health Sciences University, Ras Al Khaimah P.O. Box 11172, United Arab Emirates; 2 Geeta Institute of Pharmacy, Geeta University, Panipat 132145, India; 3 Amity Institute of Pharmacy, Amity University, Gurgaon 122413, Indiassharma@ggn.amity.edu (S.S.); 4 Department of Pharmacy, Jagannath University, Bahadurgarh 124507, India; 5 Royal Cornwall Hospital Trust, National Health Service, Truro TR1 3LJ, UK; 6 Department of Health Promotion, Mother and Childcare, Internal Medicine and Medical Specialties, School of Medicine, University of Palermo, 90127 Palermo, Italy

**Keywords:** obesity, metabolic syndrome, GLP-1 RAs, cardiovascular, injectable, oral semaglutide, SNAC, PIONEER program, type 2 diabetes, chronic kidney disease, oral peptide, formulation shifting

## Abstract

The obesity–metabolic syndrome–diabetes continuum is driven by interconnected mechanisms including insulin resistance, dysfunctional adiposity, chronic inflammation and progressive cardio–renal–metabolic injury. This triggered a need for therapies that extend beyond glucose lowering alone. The benefits of glucagon-like peptide-1 receptor agonists (GLP-1 RAs) as disease-modifying drugs include weight loss, cardiovascular risk reduction, glycemic control and renal protection. However, treatment burden, adherence issues and access restrictions may limit the long-term effects of injectable formulations. One significant translational development that aims to close this gap is oral GLP-1-based treatments. In this review, we examine the mechanistic rationale, formulation science and clinical development of oral GLP-1 RAs. Oral semaglutide is presented as the first validated proof of concept for systemic peptide delivery by the gastrointestinal route. The biological barriers to oral peptide absorption, including enzymatic degradation, low epithelial permeability, pharmacokinetic variability and epithelial safety constraints, are critically discussed. Enabling technologies such as SNAC-based gastric absorption, nanocarriers, mucoadhesive systems and stability-optimization platforms are evaluated. Evidence from the PIONEER program and related studies demonstrating meaningful glycemic and weight-loss efficacy, acceptable safety and clinical utility in patients with type 2 diabetes and chronic kidney disease is further synthesized. Beyond first-generation oral peptide platforms, we discuss the emerging landscape of non-peptide oral GLP-1 RAs, dual and triple incretin agonists, precision dosing strategies and model-informed drug development. Oral GLP-1-based therapeutics are shifting from a formulation breakthrough to a broader translational strategy for disease modification across the obesity–metabolic syndrome–diabetes continuum. Long-term renal outcomes, access and implementation barriers remain important priorities for future research.

## 1. Introduction

The cardio–renal–metabolic continuum encompassing chronic kidney disease (CKD), type 2 diabetes mellitus (T2DM), and obesity is driven by interconnected mechanisms, including insulin resistance, glomerular hyperfiltration, endothelial dysfunction, chronic inflammation, and maladaptive neurohormonal activation [1]. Persistent cardiometabolic injury is further sustained by mitochondrial dysfunction, renin–angiotensin–aldosterone system activation, and abnormalities in substrate handling. Despite advances in glucose-lowering and antihypertensive therapies, substantial residual cardiovascular and renal risk remains, highlighting the need for strategies that target multisystem disease biology rather than isolated metabolic endpoints [2,3].

GLP-1 RAs have emerged as key agents due to their pleiotropic effects beyond glycemic control. In addition to glucose-dependent insulinotropic actions, GLP-1 receptor signaling modulates inflammatory pathways, reduces oxidative stress, improves endothelial nitric oxide bioavailability, suppresses glucagon secretion, and promotes satiety. Experimental evidence shows attenuation of tubular inflammation, reduced albuminuria, and inhibition of mesangial expansion [4,5]. These effects contribute to weight reduction, decreased cardiovascular events, and slower renal disease progression, supporting their role as disease-modifying therapies [6].

Drug delivery also influences therapeutic efficacy. Although injectable GLP-1 RAs provide consistent pharmacokinetic exposure, treatment burden, injection aversion, and cold-chain requirements can limit long-term use [7]. Real-world discontinuation rates often exceed those observed in clinical trials, indicating a gap between efficacy and effectiveness. In chronic conditions, the route of administration plays a key role in long-term adherence and therapeutic impact [8,9].

A major translational advance addressing these limitations is the development of oral GLP-1–based therapies. Systemic peptide delivery via the gastrointestinal tract has been enabled by co-formulation of semaglutide with sodium *N*-[8-(2-hydroxybenzoyl) amino] caprylate (SNAC), an absorption enhancer [10,11,12,13]. This approach protects against enzymatic degradation, increases epithelial permeability, and modulates local pH to promote gastric absorption. However, limitations such as low bioavailability, luminal dilution, variability in gastric motility, and restricted absorptive surface area continue to affect systemic exposure [14].

**Figure 1:**
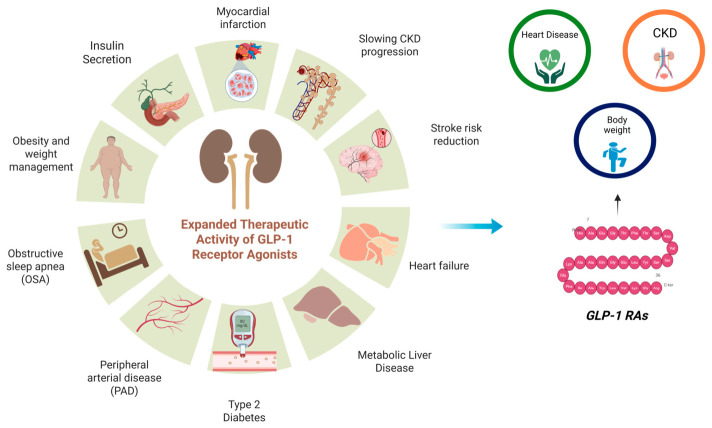
Pleiotropic cardio–renal–metabolic effects of GLP-1 RAs across organ systems. GLP-1 RAs help reduce weight, protect the heart, support kidney function, and calm inflammation throughout the body. They lower the risk of major cardiovascular events, slow kidney disease, reduce heart failure, and may even benefit liver disease and sleep apnea.

Oral administration represents a shift in therapeutic strategy, where drug delivery influences long-term disease modification (Figure 1). To address limitations of peptide delivery, non-peptide small-molecule GLP-1 receptor agonists are being developed. These agents interact with transmembrane receptor domains and may improve oral bioavailability without absorption enhancers or strict dosing conditions [15]. Their potential advantages in receptor stability, signaling and manufacturing may further reshape incretin therapy. This evolving landscape reflects a transition toward formulation-enabled precision therapy [16,17].

This review examines the mechanistic basis, biological constraints, formulation strategies and renal–cardiometabolic implications of oral GLP-1–based therapies. It also evaluates key translational challenges including pharmacokinetic variability and scalability, and highlights their potential in improving adherence, accessibility and disease outcomes across the cardio–renal–metabolic continuum.

## 2. Pharmacological Basis of GLP-1 RAs

### 2.1. Renal Protection and Weight Loss

GLP-1 RAs are incretin-based medications initially developed for type 2 diabetes mellitus and are now recognized for their metabolic and organ-protective effects. In hyperglycemic conditions, they suppress glucagon release from α-cells and enhance glucose-dependent insulin secretion from pancreatic β-cells by mimicking endogenous GLP-1 [18]. This glucose-dependent mechanism improves glycemic control while reducing the risk of hypoglycemia. GLP-1 RAs also act centrally to reduce appetite and delay gastric emptying, leading to sustained and clinically meaningful weight loss [19].

In obesity-associated diabetes, these effects are particularly important, as excess adiposity drives insulin resistance, inflammation, and kidney injury [19,20]. GLP-1 RAs-mediated weight loss is associated with improvements in blood pressure, lipid profile, and inflammatory markers that support renal protection. In addition, GLP-1 receptor activation may exert direct renoprotective effects through modulation of intrarenal hemodynamics and cellular stress pathways, beyond indirect metabolic effects. This suggests that renal benefits are not completely dependent on weight loss or glycemic improvement [16].

**Figure 2:**
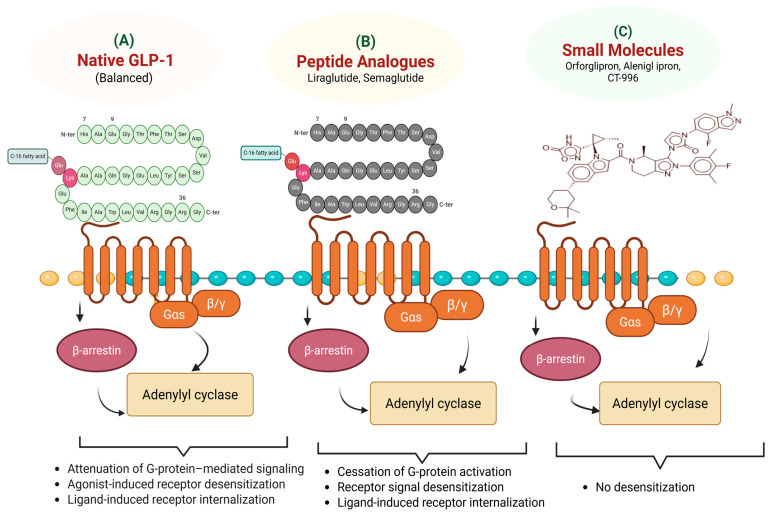
Comparative signaling profiles of native GLP-1 (**A**), peptide analogs (**B**), and small-molecule (**C**) GLP-1 Ras. Endogenous GLP-1 promotes β-arrestin activity and balanced Gαs-cAMP signaling regulated by fast receptor internalization. This is countered by peptide analogs such as semaglutide which maintain insulinotropic and anorectic signaling while varying desensitization kinetics through prolonged receptor residency and DPP-4 resistance. By binding certain transmembrane pockets, small-molecule agonists may provide biased agonism that favors prolonged G protein activation with reduced internalization, perhaps lowering tachyphylaxis and enhancing long-term receptor responsiveness.

Recent studies also indicate that GLP-1 RAs influence adipose tissue physiology by improving insulin sensitivity and reducing lipotoxic signaling. Changes in adipokine profiles, including increased adiponectin and reduced leptin resistance, may contribute to decreased renal inflammation and fibrosis. This systemic metabolic modulation supports the role of GLP-1 RAs in the broader cardio-renal axis [20]. However, the relative contribution of weight loss versus direct receptor-mediated renal effects remains unclear and requires further clinical evaluation. The receptor interactions and downstream signaling properties of native GLP-1, peptide analogs, and small-molecule agonists are shown in Figure 2.

### 2.2. Anti-Inflammatory, Natriuretic and Cardiovascular Effects

GLP-1 RAs influence renal and cardiovascular physiology beyond metabolic control. GLP-1 receptors in the proximal tubule inhibit the sodium–hydrogen exchanger 3 (NHE3), promoting natriuresis and reducing intraglomerular pressure [21]. These changes reduce glomerular hyperfiltration, an early feature of DKD. Clinically, an initial decline in estimated glomerular filtration rate (eGFR) is often observed, reflecting normalization of hyperfiltration rather than nephrotoxicity, and is associated with long-term renal benefit [22].

GLP-1 RAs also modulate inflammatory and oxidative pathways linked to CKD progression. Reductions in oxidative stress, pro-inflammatory cytokine signaling, and endothelial dysfunction have been consistently reported [23]. These mechanisms are relevant in DKD, where microvascular injury is driven by chronic inflammation. These renal effects occur alongside cardiovascular benefits, supporting the role of GLP-1 RAs as integrated cardio–renal–metabolic therapies [24,25].

Mechanistic studies suggest that GLP-1 receptor activation reduces reactive oxygen species (ROS) production in renal tubular cells and improves mitochondrial function, limiting cellular injury during hyperglycemia. Interactions between GLP-1 signaling and endothelial nitric oxide pathways may improve vascular function and reduce arterial stiffness, contributing to renal and cardiovascular protection [25].

Beyond classical cAMP-mediated signaling, GLP-1 receptor activation involves complex receptor pharmacology, including biased agonism and dynamic receptor trafficking. Different GLP-1 RAs can preferentially activate distinct intracellular signaling pathways, a phenomenon known as biased signaling, which may influence therapeutic outcomes. In addition, receptor internalization, recycling, and desensitization regulate the duration and magnitude of receptor signaling. Peptide-based agonists and emerging small-molecule agonists differ in their binding interactions, signaling bias and receptor trafficking behavior, which may contribute to differences in efficacy, tolerability, and pharmacological profiles [25,26]. These effects support a multi-organ mechanism of action rather than single-pathway regulation (Figure 1).

### 2.3. Pharmacokinetic Challenges

GLP-1 RAs have favorable pharmacodynamics but present pharmacokinetic challenges, particularly in CKD and DKD. Most long-acting GLP-1 RAs are degraded via proteolytic pathways rather than renal excretion. However, disease-related changes in gastrointestinal physiology, including altered permeability, gastric motility, systemic inflammation, and protein binding, can introduce inter-individual variability in drug exposure [26,27,28].

For oral GLP-1 formulations, therapeutic response depends on gastrointestinal absorption. Variability in luminal dilution, gastric emptying, and epithelial transport affects systemic drug levels, influencing efficacy and tolerability. Thus, long-term effectiveness depends on both pharmacological activity and consistent drug exposure. Parenteral formulations provide stable exposure but may be limited by adherence and logistical challenges [27]. The development of oral GLP-1 RAs represents an important step to improve accessibility and long-term use [28,29].

Model-informed drug development approaches, including physiologically based pharmacokinetic (PBPK) modeling, are increasingly used to predict exposure variability and optimize dosing in heterogeneous populations such as CKD. These methods are expected to support the development of next-generation oral incretin therapies [22].

**Figure 3:**
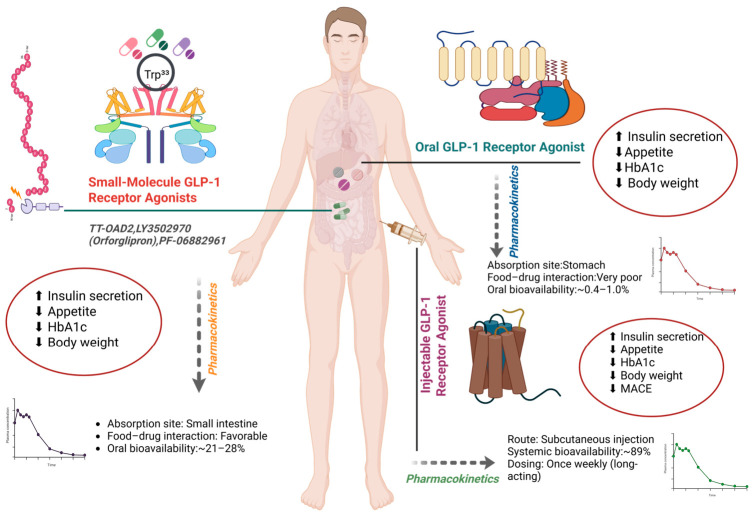
Comparative pharmacologic architecture and pharmacokinetic profiles of injectable, oral peptide and small-molecule GLP-1 RAs. ⬆-Increase, ⬇-Decrease.

To address limitations such as enzymatic degradation and low epithelial permeability, formulation design is a key determinant of clinical performance. The integration of formulation science and clinical pharmacology influences the therapeutic efficacy of oral GLP-1 therapies [29]. Figure 3 illustrates structural and pharmacokinetic differences between small-molecule GLP-1 RAs, injectable peptide analogs and absorption enhancer–based oral peptides.

## 3. Parenteral GLP-1 Formulations: Current Landscape

Developments in formulation design for intrinsic instability and enzymatic breakdown of natural GLP-1 have been crucial to the clinical success of GLP-1 RAs [11]. The short half-life of native GLP-1 was improved by the introduction of structurally altered peptides that could sustain receptor activation. A wide variety of injectable formulations have been created since GLP-1 RAs were first used, allowing for more dosage flexibility and better therapeutic coverage [29].

An important development in incretin-based therapy was the approval of exenatide in 2005 as the first-in-class GLP-1 RA with twice-daily subcutaneous dosing [30,31]. During the next ten years, the class evolved rapidly with longer-acting agents such as liraglutide, lixisenatide, albiglutide, dulaglutide and semaglutide (Table 1). These developments not only improved glycemic control but also expanded the therapeutic role in obesity management and cardiovascular risk reduction [32,33]. Despite these developments, the injectable paradigm scalability as a long-term population-level intervention is still limited by its mode of administration.

### 3.1. Marketed Injectable GLP-1 RAs (Table 1)

Currently available GLP-1 RAs can be broadly categorized based on dosing frequency, i.e., daily and once-weekly formulations. Daily agents such as liraglutide and lixisenatide provide prolonged activation of the receptor. However, due to the increased number of total daily doses, there could be a decreased rate of adherence over time. On the other hand, once-weekly formulations such as albiglutide, semaglutide, and dulaglutide may increase treatment durability and convenience [36,37,38].

A significant advancement in formulation design is the transition from daily to weekly dosing for a better therapeutic regimen in managing chronic disease [47]. Even though the reduced frequency of dosing makes it more convenient, it still will not eliminate the practical and emotional challenges of injectable therapy completely. The problems of needle fear, treatment exhaustion and disruption to one’s lifestyle are still largely unresolved [48,49].

This demonstrates that although pharmacokinetic optimization is crucial, patient-centered factors including ease of administration, therapeutic perception and long-term acceptability continue to be significant limiting factors [47].

### 3.2. Formulation Strategies: Depot Systems, PEGylation, Albumin Binding (Table 2)

Newer GLP-1 RAs have a longer half-life due to advanced drug formulation and molecular engineering techniques. These include sustained-release depot systems, PEGylation, fusion technologies and fatty acid acylation for reversible albumin binding (Table 2) [45,48,49].

Fatty acid acylation allows for reversible binding to serum albumin, reducing renal clearance and protecting the peptide from enzymatic breakdown as shown by liraglutide and semaglutide [50]. This approach has proven to be highly effective in maintaining constant plasma concentrations and prolonging half-life [51,52]. Albumin fusion and Fc-fusion technologies increase systemic exposure.

Dosing frequency can be decreased by using depot-based delivery systems such as microsphere formulations, which enable the active component to be gradually released from the injection site over a period of time. These approaches have greatly enhanced pharmacokinetic characteristics but have added manufacturing, stability and injection-site tolerability issues. These strategies highlight a translational limitation and increasing molecular complexity [53].

**Table 2:** Formulation engineering strategies for injectable GLP-1 RAs.

Formulation Strategy	Molecular Mechanism	Pharmacokinetic Impact	Clinical Advantages	Translational Limitations	Refs.
Immediate-Release Peptide	Native or minimally modified peptide	Rapid absorption; short plasma half-life	Rapid onset of glycemic effect	Frequent dosing; proteolytic degradation; low persistence	[54,55,56]
Fatty-Acid Acylation (e.g., liraglutide, semaglutide)	Reversible albumin binding via lipid side chain	Prolonged half-life; reduced renal clearance	Once-daily or weekly dosing; improved durability	Precise structural optimization required to preserve receptor affinity	[57,58,59,60,61,62]
Albumin Fusion (e.g., albiglutide)	Genetic fusion to albumin increases molecular size	Reduced renal filtration; extended systemic exposure	Once-weekly administration	Large molecular size limits tissue diffusion; potential immunogenicity	[63,64,65,66,67,68]
Fc-Fusion Technology (e.g., dulaglutide)	Fusion to IgG Fc domain increases size and stability	FcRn-mediated recycling prolongs half-life	Stable weekly pharmacokinetic profile	Complex biologic manufacturing; structural stability considerations	[69,70,71]
Depot/Sustained-Release Systems	Subcutaneous microsphere or matrix-based slow release	Gradual systemic exposure over days to weeks	Reduced injection frequency	Local injection-site reactions; formulation complexity; scalability challenges	[72,73,74]

### 3.3. Clinical Outcomes and Real-World Adherence Limitations

Despite its proven advantages, injectable therapy adherence in standard clinical practice is still low due to high dropout rates. The responsible factors include treatment tiredness, needle aversion and the practical difficulties in long-term self-administration [75,76,77]. Tolerability and persistence are also impacted by gastrointestinal side effects such as nausea and vomiting. Its discontinuation rates are higher in the real world than in clinical trials, which highlights a practical limitation in the management of chronic diseases [78].

In addition, injectable formulations require cold-chain storage, rigorous sterility conditions and sophisticated production methods, all of which contribute to greater costs and restricted accessibility, particularly in resource-limited areas [79,80].

Together, these elements lead to a gap between clinical efficacy and practical efficacy. So, the route of delivery has become a crucial factor in determining the efficacy of long-term treatment. In this regard, a paradigm shift to oral GLP-1 formulation platform is needed to increase therapeutic durability and expand availability across various healthcare units rather than just as a convenience-driven innovation [81,82,83,84].

The development of oral semaglutide represents a logical progression in addressing these challenges by improving patient acceptance and simplifying long-term therapy [85,86].

## 4. Scientific Barriers to Oral Delivery of Peptide Drugs

### 4.1. Bioavailability Constraints

The detrimental physiologic and pharmacokinetic factors that severely limit the oral delivery of peptide therapeutics include enzymatic degradation in the gastrointestinal tract. The peptide core is rapidly hydrolyzed by stomach acid, proteases and peptidases leading to a reduction in absorption [87,88,89]. In addition, poor intestinal permeability due to high molecular weight, hydrophilicity and limited transcellular diffusion of peptides across the intestinal epithelium is limited by the mucus layer [90].

Patients with CKD and DKD experience these difficulties due to several factors such as changes in gastric pH, delayed gastric emptying and intra-patient variability in intestinal transit, which leads to variable systemic absorption [34,91]. This non-linear relationship between dose and systemic absorption represents a fundamental pharmacokinetic ceiling that distinguishes oral peptide delivery from conventional small-molecule therapeutics.

Various strategies have been attempted to overcome these obstacles, such as absorption enhancers, enzyme inhibitors and structural peptide modifications (i.e., cyclization, lipidation and PEGylation). The most reliable clinical translation among these has been shown by absorption-enhancing techniques [92,93]. This approach includes co-formulation of oral semaglutide with sodium *N*-(8-[2-hydroxybenzoyl] amino) caprylate (SNAC), which minimizes exposure to intestinal breakdown while enabling transitory transcellular absorption across the gastric mucosa [13,94,95]. Despite these developments, the absolute bioavailability of oral peptides is still incredibly low (<1%), highlighting the intrinsic inefficiency of gastrointestinal peptide transport. These constraints highlight that oral peptide delivery operates within a tightly restricted biopharmaceutical window, where small physiological variations can result in disproportionate changes in systemic exposure [90].

### 4.2. Translational Constraints of SNAC-Enabled Gastric Absorption

Despite demonstrated clinical effectiveness, important translational constraints remain in SNAC-mediated delivery. The bioavailability of oral semaglutide remains below 1%, reflecting the limited absorptive capacity of the gastric epithelium [88,96]. Increasing the dose does not result in proportional systemic exposure, indicating a non-linear absorption profile. This limitation is closely linked to the dependence of absorption on gastric residence time. Variations in drug dispersion and retention therefore contribute to inconsistent systemic exposure.

Structural factors further restrict absorption. Mucus thickness and limited epithelial transport reduce the ability of higher doses or increased enhancer concentrations to improve uptake. As a result, dose intensification strategies are inherently constrained. Clinically, this suggests that increasing dose alone may not reliably overcome absorption limitations [97].

Inter-individual variability further complicates this delivery approach. The efficiency of SNAC-mediated absorption depends on physiological factors such as fasting state, gastric pH, motility, and the positioning of the dosage form within the stomach. In patients with CKD, this variability may be amplified by autonomic dysfunction, delayed gastric emptying, uremic mucosal alterations, and concomitant medications that affect mucosal physiology [98,99]. Consequently, variability in drug absorption may translate into variability in therapeutic response, particularly in heterogeneous patient populations. Although dose titration may reduce short-term fluctuations, persistent long-term variability remains a concern. This is especially relevant for renal outcomes, where sustained receptor activation is required [100,101].

Epithelial biology represents another critical consideration. SNAC facilitates transcellular transport by transiently altering membrane fluidity and locally buffering gastric pH without permanently disrupting tight junction integrity. However, the long-term effects of repeated exposure to high local concentrations of both the drug and the enhancer remain uncertain [102]. This highlights a central challenge in oral peptide delivery, achieving sufficient permeability while preserving epithelial integrity. Preclinical findings suggest the possibility of reversible micro-injury, raising concerns about cumulative epithelial stress during prolonged therapy [103,104,105]. Clinically, this implies that dose escalation alone may not reliably overcome absorption limitations, reinforcing the need for next-generation delivery platforms.

## 5. Enabling Technologies for Oral GLP-1 Formulations

From a biopharmaceutical perspective, oral semaglutide can be considered analogous to a Biopharmaceutics Classification System (BCS) class IV compound, exhibiting both low solubility and low permeability. These properties, combined with enzymatic degradation in the gastrointestinal tract, contribute to its limited and variable oral bioavailability [104]. Enabling technologies for oral GLP-1 formulations are as follows.

### 5.1. Absorption Enhancers

**Figure 4:**
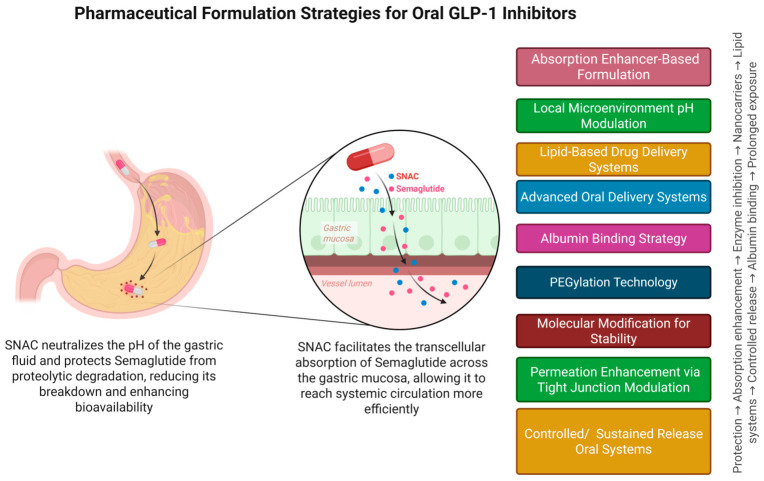
Pharmaceutical formulation strategies for oral GLP-1 receptor agonists: mechanistic basis and delivery platform overview. The diagram illustrates how semaglutide is a prime example of how co-formulation with SNAC provides a solution by two mechanisms, i.e., by temporarily increasing transcellular permeation across the gastric epithelium, which results in significant systemic bioavailability and by locally alkalinizing the gastric microenvironment to reduce proteolytic degradation.

The SNAC-based drug delivery system is one of the most innovative oral GLP-1 delivery platforms currently available. SNAC locally and reversibly alters the gastric microenvironment, thus increasing transcellular transport through the gastric epithelium and temporarily increasing pH [104,105,106,107,108]. It reduces the exposure of the drug to proteolytic enzymes and extensive intestinal permeation by promoting absorption in the stomach rather than in the intestine. SNAC preserves the natural structure and pharmacological activity of semaglutide by not forming covalent complexes with the peptide (Figure 4) [109].

As discussed above, SNAC-mediated delivery operates within a narrow absorption window and is associated with low and variable bioavailability [110]. Absorption enhancers only temporarily circumvent epithelial barriers rather than completely overcoming them; their effectiveness is greatly dependent on local physiological conditions [16,103].

Medium-chain fatty acids, bile salts and surfactants are among the other groups of permeation enhancers that have been studied to facilitate epithelial transport. Although these compounds can improve permeability, safety issues such as mild epithelial irritation and long-term barrier disruption have restricted their clinical translation [111,112,113]. These are currently the most sophisticated approaches that are under research for their long-term safety and scalability.

### 5.2. Nanocarriers and Permeation Enhancers

Nanocarriers, such as polymeric nanoparticles, lipid nanoparticles and hybrid nanostructures, are designed to protect peptides from enzyme degradation, enable controlled release and improve epithelial transport [113]. These systems enhance stability in the gastric environment which is crucial for CKD patients having altered gastric physiology [114].

**Figure 5:**
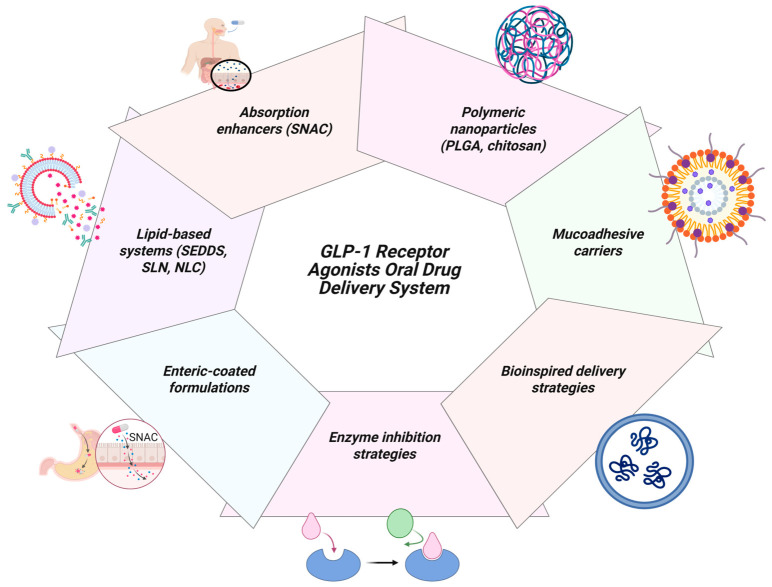
Formulation strategies enabling the oral delivery of GLP-1 RAs. The schematic shows key formulation approaches including absorption enhancers such as SNAC and polymeric nanoparticle-based systems (e.g., PLGA, chitosan), designed to overcome gastrointestinal barriers such as enzymatic degradation and limited epithelial permeability, thereby improving systemic bioavailability of peptide drugs.

Lipid-based systems such as solid lipid nanoparticles, liposomes and self-emulsifying drug delivery systems (SEDDS) utilize endogenous lipid absorption mechanisms to bypass first-pass metabolism and enhance lymphatic uptake [115]. These mechanisms improve gastrointestinal residence time and enhance transcellular transport. However, clinical validation for GLP-1 RAs is limited, while lipid formulations offer advantages of formulation flexibility and scalability and thus present an attractive translational opportunity for oral GLP-1 RAs [93,116]. Multiple formulation approaches have been developed to address gastrointestinal barriers; these approaches—each targeting distinct limitations in peptide stability and permeability—are depicted in Figure 5.

Surface functionalization techniques such as PEGylation or ligand targeting have been employed to enhance mucoadhesion or receptor-mediated uptake [117]. Polymers such as carbomers, thiolated polymers, and chitosan derivatives can interact with the mucus layer to increase paracellular permeability and epithelial residence durations. Although these approaches significantly improve stability and absorption in preclinical models, their safety remains a challenge [118,119].

There are significant limitations associated with the production of nanocarrier systems at a large scale, including batch-to-batch reproducibility and long-term safety, which remain hurdles in clinical translation. Structural and functional complexities of nanocarrier systems are additional complexities during the regulatory process, making it more complicated and expensive [113].

### 5.3. Mucoadhesive and Targeted Delivery Systems

Mucoadhesive delivery systems aim to extend residence time at the absorption site by adhering to the gastrointestinal mucosa [114,120]. Polymers like chitosan and carbopol are used to enhance adhesion and facilitate paracellular transport. Targeted delivery strategies utilize receptor-mediated uptake mechanisms by functionalizing carriers with specific ligands that attach to epithelial transport proteins. Despite their advantages, these approaches have a number of real-world drawbacks [92,121]. Their gastrointestinal mucosal contact is limited by luminal flow, peristalsis and constant mucus turnover in the highly dynamic gastrointestinal environment. Therefore, the theoretical advantages of longer residence times may not translate into consistent in vivo absorption. Physiological variability nevertheless restricts the practical efficacy of targeted and mucoadhesive techniques despite their mechanistic advantages [122,123].

### 5.4. Device-Based and Mechanical Delivery Systems

An alternative technique for delivering oral peptides through devices provides a new approach to bypassing the epithelial barriers. These include ingestible devices that remain in the stomach, microneedle delivery systems and self-orienting delivery systems that deliver peptides into the gastric and intestinal wall tissues [92,120].

Although device-based solutions may overcome the basic bioavailability restriction associated with traditional oral peptide delivery, they introduce additional difficulties such as patient acceptability, manufacturing complexity, device safety and regulatory issues. A major formulation engineering problem is the need for mechanical precision and reliability in a highly challenging gastrointestinal environment [123].

An emerging approach in device-based oral peptide delivery is the self-orienting millimeter-scale applicator (SOMA) system, which is designed to enable direct drug delivery across the gastric mucosa. This ingestible device utilizes a self-orienting mechanism to position a microneedle against the stomach wall, allowing localized injection of peptide therapeutics such as insulin. By bypassing the gastrointestinal barriers associated with enzymatic degradation and poor permeability, SOMA offers a novel strategy to achieve systemic delivery of macromolecules via the oral route. Preclinical studies have demonstrated its feasibility and potential for improving bioavailability, although further clinical validation is required to establish safety, scalability and long-term applicability [92].

### 5.5. Small-Molecule Oral GLP-1 RAs

In parallel with peptide-based approaches, small-molecule oral GLP-1 RAs have emerged as an alternative strategy with distinct pharmacological and translational advantages. Unlike peptide formulations, which rely on absorption enhancers and exhibit low and variable bioavailability, small-molecule agonists generally demonstrate improved oral absorption, fewer dosing restrictions and greater formulation flexibility. From a pharmacological perspective, these agents may exhibit differences in receptor binding, signaling bias and duration of action compared with peptide-based agonists [122].

In addition, small-molecule platforms offer potential advantages in large-scale manufacturing, stability, and distribution, supporting improved scalability and global accessibility. However, their long-term efficacy, receptor selectivity, and safety profiles remain under active investigation, and direct comparisons with established peptide-based therapies are still limited. A critical evaluation of these differences is essential for guiding the development of next-generation GLP-1 RAs [122].

### 5.6. Comparative Translational Perspective

A comparative analysis of existing technologies shows that each strategy represents a trade-off between bioavailability, variability, safety and scalability; no single platform completely removes the basic obstacles to oral peptide administration.

According to this comparison, absorption-enhancer-based systems will likely be the most popular platform in the near future. The other sophisticated technologies might need to make significant advancements in engineering and regulatory science before they can be widely used. In order to maximize efficiency and safety, future innovation may rely on hybrid systems that combine several strategies, such as combining efficacy with targeted delivery [124].

An additional advantage of oral GLP-1 formulations is the potential to reduce reliance on cold chain storage. This can lead to easier accessibility, reduce overall logistics costs and improve patient adherence in low-resource areas [125]. The oral GLP-1 platform success will depend on an ideal balance between efficacy, stability, safety and practical usability rather than just focusing on bioavailability.

## 6. Oral GLP-1 RAs: Clinical Evidence

### 6.1. PIONEER Program: Efficacy and Safety

The PIONEER trial program, a comprehensive set of randomized controlled trials assessing the efficacy and safety of oral semaglutide across a variety of patient populations with type 2 diabetes mellitus (T2DM), has been largely responsible for the clinical development of this medication. Collectively, these trials demonstrate that oral semaglutide significantly reduced body weight, cardiometabolic risk factors and glycated hemoglobin (HbA1c) in all of these investigations [126,127,128,129].

Across individual studies, oral semaglutide consistently showed superior glycemic control compared with placebo and empagliflozin (PIONEER 1 and 2), non-inferiority to subcutaneous liraglutide (PIONEER 4), and dose-dependent efficacy relative to sitagliptin (PIONEER 3), with parallel improvements in body weight (Table 3) [98,130,131]. These findings indicate that, despite its low oral bioavailability, semaglutide retains the pharmacodynamic effects characteristic of GLP-1 receptor activation.

These findings demonstrate that despite its poor bioavailability, oral semaglutide maintains the essential pharmacodynamics of GLP-1 receptor activation. Oral semaglutide’s safety profile is mostly similar to that of injectable GLP-1 RAs, with the most common reported side effects being gastrointestinal, mainly nausea, vomiting and diarrhea [132]. These effects are dose-dependent and decrease with time when followed by correct dose escalation. However, the use of strict dosage requirements, including fasting state, restricted water intake and delayed food eating, adds another level of complication that could affect adherence in the real-world.

The interpretation of the PIONEER findings should consider inter-trial heterogeneity and differences relative to injectable GLP-1 RA trials such as the SUSTAIN program. Variations in study design, including dosing frequency (oral daily versus subcutaneous weekly), comparator selection, and trial duration, as well as differences in patient populations and baseline cardiovascular risk, limit direct cross-trial comparisons. Furthermore, the controlled conditions of clinical trials may overestimate treatment effectiveness, particularly given the strict dosing requirements and potential pharmacokinetic variability associated with oral semaglutide. Therefore, comparisons between oral and injectable formulations should be interpreted cautiously within the context of these methodological differences [98,127,128,129]. Therefore, while trial efficacy is robust, its translation into routine clinical practice may be attenuated by adherence constraints and pharmacokinetic variability.

### 6.2. Cardiovascular and Renal Outcomes

GLP-1 RAs have shown significant effects in cardiovascular outcome trials (CVOTs) in lowering major adverse cardiovascular events (MACE). The PIONEER 6 study demonstrated the cardiovascular safety of oral semaglutide, indicating a trend toward reduction in MACE when compared to a placebo [138,139]. The results were similar to those for injectable semaglutide in the SUSTAIN-6 trial. Shorter trial durations and lower event rates may mask oral formulations’ true cardiovascular potential. Large-scale trials like SOUL are planned to assess long-term cardiovascular outcomes with oral semaglutide in higher-risk individuals and may provide more conclusive evidence [140,147].

Beyond cardiovascular effects, GLP-1 RAs have shown favorable renal outcomes, including reductions in albuminuria, attenuation of estimated glomerular filtration rate (eGFR) decline, and delayed progression to macroalbuminuria. While these benefits are well established for injectable agents, it remains uncertain whether oral formulations can achieve sufficiently consistent systemic exposure to replicate comparable long-term renal protection. This distinction underscores the importance of sustained pharmacokinetic exposure in mediating organ-protective effects, beyond short-term efficacy alone [141,147].

### 6.3. Real-World Evidence and Adherence

While randomized controlled trials provide robust evidence of efficacy and safety, real-world data are essential for understanding treatment performance in routine clinical practice. Early real-world studies suggest that oral semaglutide improves treatment acceptance compared with injectable therapy, particularly among patients reluctant to initiate injections or those with needle aversion [135].

However, adherence remains influenced by the unique administration requirements of oral semaglutide. These include fasting conditions, limited water intake, and delayed food consumption. Such constraints introduce behavioral complexity that may offset some of the convenience typically associated with oral medications [76,142]. As a result, oral semaglutide improves acceptability but does not fully resolve adherence challenges.

Discontinuation rates remain clinically relevant in real-world settings. Adherence is influenced by gastrointestinal intolerance, dosing complexity, and patient expectations. In this context, pharmacological efficacy alone is insufficient to ensure long-term treatment success. Behavioral support and patient education remain critical determinants of persistence [143].

Importantly, clinical trials such as the PIONEER and SUSTAIN programs are conducted under controlled conditions, with structured dose escalation and close monitoring. These conditions may overestimate treatment effectiveness compared with routine practice. In contrast, real-world settings are characterized by variable adherence, heterogeneous populations, and less intensive follow-up, all of which can attenuate therapeutic outcomes. Therefore, trial findings should be interpreted within the context of real-world implementation, particularly when evaluating long-term effectiveness and treatment durability.

### 6.4. Comparative Perspective: Oral vs. Injectable GLP-1

A key question in clinical translation is whether oral GLP-1 therapies can match the efficacy and outcome benefits of injectable formulations. Head-to-head comparisons, such as PIONEER 4, suggest that oral semaglutide achieves glycemic control comparable to injectable liraglutide (Table 4). However, this apparent equivalence should be interpreted with caution.

Injectable GLP-1 receptor agonists provide consistent and predictable pharmacokinetic exposure due to near-complete systemic bioavailability. In contrast, oral semaglutide is characterized by low and highly variable absorption that depends on strict dosing conditions, including fasting state and gastric physiology. This variability may lead to less consistent therapeutic responses in real-world settings, despite similar efficacy observed under controlled trial conditions [143].

In addition, injectable formulations are supported by robust cardiovascular outcome data, whereas long-term outcome evidence for oral GLP-1 RAs remains comparatively limited. Although oral therapy improves treatment acceptability by eliminating injections, it introduces adherence and exposure-related challenges, particularly due to stringent dosing requirements [132,144]. As a result, convenience is not absolute and may vary across patient populations.

Clinical decision-making therefore requires balancing pharmacokinetic reliability and outcome evidence against patient preference and treatment acceptability. Cross-trial comparisons between oral (PIONEER) and injectable (SUSTAIN) programs should be interpreted cautiously, as differences in study design, patient populations, and dosing strategies limit direct comparisons [145].

From a clinical perspective, oral GLP-1 RAs may facilitate earlier intervention, particularly in patients who are unwilling or reluctant to initiate injectable therapy [145,146]. In contrast, injectable formulations remain preferable in advanced disease, where consistent and sustained receptor activation is required.

Overall, oral GLP-1 RAs should not be viewed as direct substitutes for injectable therapies. Instead, they represent complementary options within a stratified treatment framework based on disease stage, patient preference, and the need for pharmacokinetic consistency. In parallel, emerging small-molecule GLP-1 receptor agonists may further expand this landscape by offering improved oral bioavailability and simplified dosing, although their long-term clinical outcomes remain under evaluation [146].

The competitive landscape of Oral GLP-1 RAs is illustrated in Table 5 and a comprehensive overview of clinical trial status is discussed in Table 6.

Although Table 5 highlights the evolving competitive landscape of oral GLP-1 receptor agonists, the full spectrum of ongoing clinical development, including early and late-phase trials, is detailed in Table 6 to provide a comprehensive overview of the pipeline.

## 7. GLP-1/GIP Dual Agonists: Ongoing Clinical Trials and Translational Implications

Tirzepatide represents a major advancement in incretin pharmacology, with its single-molecule design enabling simultaneous activation of both GLP-1 and glucose-dependent insulinotropic polypeptide (GIP) receptors. Tirzepatide is structurally derived from the GIP and conjugated to a C20 fatty diacid moiety; it is administered once a week subcutaneously and has a half-life of roughly five days due to reversible albumin binding [162]. Pharmacokinetic analyses show consistent exposure across different levels of renal and hepatic impairment, reducing the need for dose modification [163].

Early-stage clinical trials showed dose-dependent decreases in body weight and glycated hemoglobin (HbA1c), which were confirmed in the SURPASS program in multiple clinical settings [164]. Tirzepatide consistently outperformed basal insulin, selective GLP-1 RAs and placebo in terms of HbA1c reduction and glycemic target attainment. Its significance as a comprehensive metabolic therapy was reinforced by significant decreases in body weight, blood pressure and lipid indices in addition to glycemic management [165,166,167].

Tirzepatide has shown therapeutic potential in several cardiometabolic categories in addition to glycemic outcomes. It improved quality of life with significant and long-lasting weight loss in obesity-focused trials (SURMOUNT program) [168]. Further research indicates positive effects on cardiac output and hepatic steatosis, such as improvements in heart failure with preserved ejection fraction (HFpEF) and decreases in liver fat content, indicating extensive systemic metabolic effects [169]. When compared with insulin glargine, post hoc analyses from SURPASS-4 show improvements in composite renal endpoints, slower declines in estimated glomerular filtration rate (eGFR), and reductions in albuminuria in renal outcomes [170]. Its renoprotective profile is anticipated to be further clarified by ongoing trials like TREASURE-CKD.

The clinical positioning of dual incretin agonists requires careful interpretation despite encouraging results. Although Tirzepatide demonstrates greater metabolic effectiveness, its drawbacks, such as gastrointestinal side effects, dose-escalation restrictions and the ongoing need for subcutaneous administration, must be taken into account [169]. Even though cardiovascular safety has been proven, conclusive proof of superiority over current GLP-1 receptor agonists is still being investigated, especially in populations already receiving optimal cardioprotective therapy [171,172].

The advent of dual agonists presents a therapeutic alternative. Oral GLP-1-based medicines mainly target practical issues, including treatment acceptance, adherence and accessibility while dual agonists seek to optimize pharmacological efficacy through multi-receptor signaling. This contrast implies that in the larger context of cardio–renal–metabolic care these strategies are probably going to be complimentary rather than competitive [173].

Extending multi-receptor incretin pharmacology to oral administration platforms is a major translational issue. Oral bioavailability is significantly hampered by the structural complexity and greater dose requirements of multiple and triple agonists, especially when peptide absorption is already limited. Therefore, future advancements may depend on the creation of non-peptide small-molecule agonists that can replicate multi-receptor activity without the requirement for absorption enhancers or stringent dosage conditions [174,175].

Other dual and triple incretin-based treatments are progressing through clinical development concurrently. While triple agonists that target GLP-1, GIP and glucagon receptors attempt to further enhance metabolic outcomes, GLP-1 and glucagon receptors agents like Mazdutide aim to combine increased energy consumption with glucose-lowering benefits. Long-term efficacy, safety and improvements to cardiovascular or renal outcomes have not yet been adequately proven, despite encouraging early-phase results [176,177,178].

**Figure 6:**
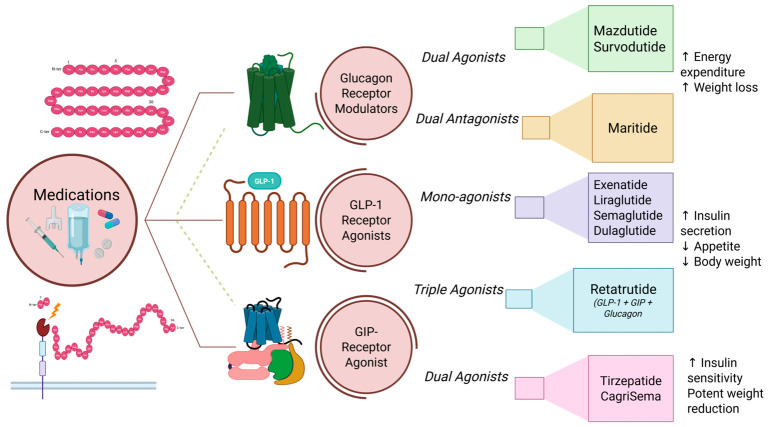
Evolution of incretin-based therapeutics: from GLP-1 receptor agonism to dual and triple hormone modulation. Early GLP-1 medications, such as semaglutide and liraglutide, were restricted to a single route and functioned by increasing insulin, decreasing glucagon, and reducing appetite. Tirzepatide and other dual agonists target two hormonal receptors at once, resulting in increased metabolic advantages and weight loss. Retatrutide and other triple agonists increase insulin sensitivity and fat burning by simultaneously affecting three pathways; ⬆-Increase, ⬇-Decrease.

GIP and glucagon pathway-based multi-receptor targeting techniques have replaced selective GLP-1 receptor activation in recent therapeutic developments. Figure 6 summarizes the growing field of incretin-based treatments, such as single-, dual-, and triple-receptor agonists.

In general, dual and newly developed triple incretin agonists signify a shift toward metabolic regulation of many pathways. However, compared to oral GLP-1 treatments, their translational trajectory is fundamentally different. The oral GLP-1 approach focuses on increasing treatment persistence and population-level impact by addressing delivery barriers and adherence challenges, whereas dual agonists emphasize maximal efficacy through improved receptor engagement. Future treatment paradigms will probably be determined by the integration of several alternative strategies [179,180].

## 8. Formulation Shift Impact on Renal Therapeutics

By providing non-injectable administration in patients with type 2 diabetes and chronic kidney disease, oral GLP-1 receptor agonists led by Semaglutide (Rybelsus^®^) represent a substantial change in incretin therapy [181]. Oral semaglutide consistently improves glycemic management and encourages weight reduction in clinical trials and real-world research [149,182]. Stable estimated glomerular filtration rate (eGFR) trajectories and decreases in the urine albumin-to-creatinine ratio (UACR) indicate that oral formulations maintain renoprotective effects similar to injectable GLP-1 RAs.

Although effects on composite renal endpoints are still being investigated, evidence from randomized studies such as the SOUL trial suggests that oral semaglutide may slow eGFR reduction in high-risk patients [183,184]. However, the current evidences for renal protection is derived from short-duration studies and surrogate markers. Long-term outcome trials are still needed to determine whether oral formulations can reproduce the complete renoprotective profile seen with injectable therapy in progressing CKD [185].

### 8.1. Pharmacokinetic Profiling

Oral semaglutide is pharmacokinetically different from many glucose-lowering medications in that renal excretion is not the primary method of elimination. Rather, it is subjected to proteolytic breakdown, which allows for a broad range of renal function based on eGFR without requiring dose modification [186]. This is particularly applicable in CKD patients, where changed renal clearance frequently calls for dose modification. However, physiological characteristics in CKD, such as stomach pH, motility and mucosal integrity, continue to have a significant impact on gastrointestinal absorption. As a result, medication exposure variability calls for cautious dose titration and clinical monitoring before starting treatment [178].

### 8.2. Adverse Effects

Therapeutic utilization of oral GLP-1 RAs in CKD populations is further influenced by adverse effect patterns. Gastrointestinal symptoms such as nausea and vomiting may be more clinically important in individuals with compromised renal function [187]. Gradual dose escalation and customized titration strategies are essential to maximize tolerability while preserving therapeutic efficacy in this population.

Another level of complexity in the treatment of CKD is polypharmacy. Oral GLP-1 RAs have the ability to affect intestinal transit and gastric emptying, which may change the absorption of co-administered drugs such as anticoagulants, calcium channel blockers and antihypertensives [188]. Although most drug–drug interaction studies report minimal clinically significant interactions with commonly used agents such as metformin and proton pump inhibitors, caution is required for drugs with narrow therapeutic indices [189]. Medication distribution and free drug concentrations may be impacted by CKD-related changes such as hypoalbuminemia and transporter activity, which emphasizes the necessity of individualized therapeutic monitoring [190].

### 8.3. Translational Prospective on Renal Therapeutics

Oral GLP-1 RAs and SGLT2 inhibitors are complementary within the broader framework of renal therapeutics. SGLT2 inhibitors exert renoprotective effects primarily through hemodynamic mechanisms, including reduction in intraglomerular pressure and restoration of tubuloglomerular feedback [190,191]. In contrast, GLP-1 RAs act mainly through metabolic and anti-inflammatory pathways. These include improved glycemic control, weight reduction, suppression of sodium–hydrogen exchanger 3 (NHE3), and reduction in oxidative stress [176,190].

Clinically, GLP-1 RAs consistently reduce albuminuria and provide broader cardiometabolic benefits. SGLT2 inhibitors, however, show stronger effects on eGFR slope stabilization [191]. This mechanistic complementarity supports combination therapy as a rational strategy, with additive benefits on glycemic control, body weight, and albuminuria. Nevertheless, the magnitude of this synergy remains uncertain, and trials powered for hard renal outcomes are still needed.

Oral GLP-1 RAs expand the clinical utility of incretin therapy by addressing barriers associated with injectable administration, particularly treatment acceptance. However, this advantage must be balanced against dosing complexity and pharmacokinetic variability. As a result, oral formulations are best viewed as an expansion of the therapeutic toolkit rather than direct substitutes for injectable agents [192].

From a clinical perspective, oral GLP-1 RAs may be particularly valuable in early-stage CKD, where long-term adherence and treatment persistence are critical. Injectable formulations remain important in advanced disease, where consistent pharmacokinetic exposure is required.

Future progress will depend on generating robust long-term renal outcome data, optimizing dosing strategies in physiologically diverse populations, and integrating oral incretin therapies into combination treatment frameworks. These steps will be essential to maximize cardio-renal protection and translate pharmacological efficacy into sustained clinical benefit [193]. Oral GLP-1 RAs should be positioned as early-intervention therapies rather than replacements for high-intensity injectable regimens in advanced disease.

## 9. Regulatory, Manufacturing, and Access Considerations

### 9.1. Regulatory Expectations for Oral Peptide Formulations

Oral peptide therapeutics represent a paradigm shift that challenges established regulatory frameworks designed for parenteral biologics or small molecules. Regulatory agencies like the US FDA and EMA demand a comprehensive demonstration of the biopharmaceutical robustness of oral peptides, including formulation-enabled absorption, gastrointestinal safety and exposure-response consistency [194]. When considering GLP-1 RAs, emphasis must be placed on identifying excipient-mediated permeability enhancement, local gastrointestinal tolerability and inter-individual variability in systemic exposure.

Regulators are expecting more and more mechanistic explanations for the use of absorption enhancers, long-term exposure evaluations, human intestinal tolerability data and nonclinical safety margins. Oral GLP-1 formulations must show bioequivalence or clinical significance in comparison to injectable therapies in terms of weight loss and cardiometabolic outcomes in addition to glycemic endpoints [195]. From a translational standpoint, regulatory submissions focus on bridging parenteral and oral formulations employing pharmacokinetic–pharmacodynamic modeling, especially in populations with renal impairment [196].

### 9.2. Scale-Up, Cost, and Cold-Chain Independence

The clinical and economic viability of oral GLP-1 RAs is heavily influenced by their manufacturing scalability. Even though peptide synthesis is a well-established process, manufacturing is further limited by the incorporation of specific excipients, intricate solid-state designs and moisture-sensitive formulations. It is necessary to establish regulatory-grade reproducibility of peptide-excipient microenvironments, content uniformity at low drug doses and long-term stability under various climatic circumstances [197].

Oral GLP-1 formulations have a significant translational advantage of cold-chain independence, which enables providers to reduce medicine waste and distribution interruptions, especially when resources are limited [198]. ICH climatic zone standards are necessary to achieve room temperature stability without compromising the integrity of the peptide using complex formulation engineering and rigid stability programs. Oral formulations offer the possibility of reducing overall cost by increasing adherence, decreasing problems caused by injections and reducing the amount of health care utilization related to parenteral administration [199].

### 9.3. Market Access and Affordability in Low- and Middle-Income Countries

Although GLP-1 RAs have been shown to have clinical utility, yet they remain largely unavailable to patients in many low- and middle-income countries due to high acquisition prices, distribution challenges and insufficient reimbursement systems. Oral formulations of GLP-1 RAs increase patient access and patient adherence based on primary care models by improving storage, transport and administrative ease [200,201,202].

Further, the affordability of oral GLP-1 RAs in these countries will depend on patient status, pricing strategies, public health and business efficiency. Fostering regional manufacturing partnerships, knowledge transfer and regional regulatory harmonization may improve access to oral GLP-1 RA therapies in these regions [203]. Diverse patient populations participating in clinical research programs, particularly those with advanced CKD or high cardiovascular and metabolic risk, will ensure these treatments have global relevance and support equitable reimbursement and regulatory decision-making for these oral therapies. The benefits of oral GLP-1 RAs will likely remain concentrated among high-income health systems, and so their overall public health benefit will be limited [204].

## 10. Future Perspectives and Emerging Trends

The therapeutic landscape of incretin-based treatments is shifting from single-pathway glucose reduction toward integrated, multifaceted metabolic modulation. The development of oral formulations represents a major advance, particularly for patients requiring long-term therapy and improved adherence. However, overcoming the inherent bioavailability limitations of peptide-based drugs remains a central challenge.

Current oral peptide platforms rely on absorption enhancers and strict dosing conditions. These constraints highlight the need for next-generation systems capable of providing more consistent systemic exposure. In this context, non-peptide small-molecule agonists represent a promising strategy. They may offer improved oral bioavailability, fewer dosing restrictions, and greater formulation flexibility by avoiding enzymatic degradation and epithelial transport barriers [205,206].

Advances in structural biology, particularly through cryo-electron microscopy, have improved understanding of receptor conformational dynamics. This has enabled the design of ligands with enhanced receptor selectivity and biased signaling profiles [207]. Such developments may lead to more effective therapies with reduced receptor desensitization and improved tolerability.

Model-informed drug development is also becoming increasingly important. Physiologically based pharmacokinetic (PBPK) modeling integrates factors such as renal function, gastrointestinal physiology, and inter-individual variability to optimize dosing strategies. This approach is especially relevant for oral peptide therapies, where systemic exposure is highly variable, and for patient populations such as those with CKD [70].

Combination therapy represents another important direction. The use of GLP-1 receptor agonists alongside SGLT2 inhibitors provides a biologically complementary approach by targeting both metabolic and hemodynamic pathways of disease progression. The development of fixed-dose oral combinations may further improve adherence and simplify treatment regimens [208].

**Figure 7:**
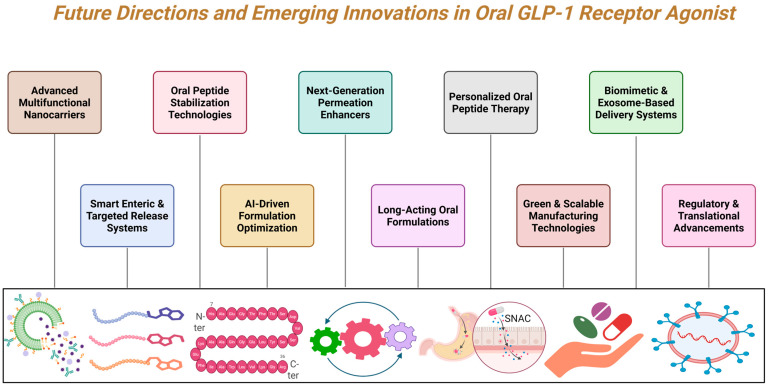
Future perspectives of oral GLP-1RAs and emerging innovation.

Looking ahead, the future of incretin therapy will be shaped by the integration of pharmacology, formulation science, and precision medicine. The goal is not only to improve bioavailability but also to achieve a balance between efficacy, safety, consistency, and real-world usability. This shift reflects a broader transition toward therapies that are both pharmacologically effective and practically sustainable for long-term disease management. Emerging technologies and future innovations in oral GLP-1 RAs are summarized in Figure 7.

## 11. Despite Substantial Progress, Critical Questions Remain: Future Research Priorities

Even though incretin pharmacology and oral peptide delivery have advanced significantly, there is still a number of unsolved scientific and translational issues, especially when it comes to renal complications. For oral GLP-1-based treatments to be sustainably included in long-term cardiometabolic and nephroprotective therapy, these gaps must be filled.

### 11.1. Long-Term Renal Outcome Data Gaps

Strong information about long-term renal effects is still lacking, particularly for medicines that are taken orally, despite the fact that numerous randomized controlled studies have shown that GLP-1 RAs enhance glycemic control, cause weight reduction, and lower cardiovascular risk. Instead of reporting hard renal outcomes like progression to end-stage kidney disease, need for renal replacement therapy, or renal mortality, the majority of available studies mainly report short- to medium-term endpoints like estimated glomerular filtration rate (eGFR) stability, albuminuria reduction, or safety in moderate CKD [209]. Patients with dialysis reliance and advanced CKD (stages 4–5) are still underrepresented in pivotal trials, which restrict the applicability of current findings to high-risk renal populations. Thus, there is an unmet need for long-term, sufficiently powered trials created especially to evaluate the course of renal disease.

### 11.2. Safety of Absorption Enhancers

The use of absorption enhancers such as SNAC has enabled the clinical translation of oral peptide therapies; however, their long-term safety profile remains incompletely characterized. While short-term clinical data suggest acceptable tolerability, preclinical studies have indicated the possibility of reversible epithelial micro-injury, raising questions regarding cumulative effects with chronic exposure [210]. Although GLP-1 receptor agonists are intended for lifelong use in many patients, comprehensive long-term safety evaluations, including post-marketing surveillance and mechanistic studies, are essential to ensure sustained epithelial integrity and a favorable benefit–risk profile.

### 11.3. Translational Hurdles from Bench to Bedside

The transition of oral peptide formulations from experimental to clinical application is linked with multiple challenges, including variability in gastrointestinal physiology, inconsistent absorption and complexities in large-scale manufacturing. The therapy response is further complicated by variations in patient-specific variables, especially in CKD patients. Furthermore, there are still concerns surrounding the approval procedures for innovative formulation technologies, and regulatory paths for complicated delivery systems are still developing [211]. It requires better preclinical models, reliable pharmacodynamic indicators and refined clinical trial designs that accurately reflect real-world variability to overcome these obstacles.

### 11.4. Unmet Clinical and Developmental Needs

The advancement of oral GLP-1 RAs depends on the capacity to create oral peptide platforms that are suitable for a broad spectrum of patients and confirm the safety of absorption-enhancing technologies for long-term usage. To fully utilize incretin therapy to alter the course of disease in individuals with metabolic and renal problems, these issues must be resolved [212].

Despite significant progress, key knowledge gaps remain in GLP-1 RAs. Long-term cardiovascular and renal outcomes for oral formulations are still limited, and real-world effectiveness may differ from clinical trial findings due to adherence and pharmacokinetic variability. Further research is needed to optimize oral delivery technologies and evaluate next-generation GLP-1-based therapies. Addressing these gaps will be essential for improving clinical translation and patient outcomes [212].

## 12. Conclusions

The development of injectable GLP-1 receptor agonists marked a significant advancement in the treatment of type 2 diabetes, obesity and DKD, with well-established benefits in glycemic control, weight reduction and cardiovascular outcomes. However, their clinical utility is limited by challenges related to administration and long-term adherence. The emergence of oral semaglutide demonstrates that systemic incretin therapy can be achieved without injections by integrating peptide stability, targeted gastric absorption and controlled systemic exposure.

From a cardiometabolic and renal perspective, oral GLP-1 RAs confer a significant reduction in metabolic risk while maintaining a favorable safety profile and a low risk of causing hypoglycemia. Their anti-inflammatory effects alongside metabolic benefits support their role in combination renoprotective strategies. In addition, oral formulations enable earlier treatment initiation and improve long-term adherence. They represent a strategic expansion rather than a replacement of incretin therapy.

Future developments are expected to focus on dual incretin agonists, model-informed precision dosing in CKD, non-peptide small-molecule agonists and rational oral combination therapies with SGLT2 inhibitors. These advances reflect a shift from formulation-focused innovation toward integrated disease modification and optimized pharmacotherapy. Oral GLP-1–based therapies are therefore likely to play an important role in the management of cardiometabolic and renal diseases.

## Figures and Tables

**Table 1:** Evolution of injectable GLP-1 RAs: formulation strategies, clinical efficacy and limitations.

Brand Name	Company	Dosing Frequency	Formulation Strategy	HbA1c Reduction (%)	Weight Reduction (kg)	Cardiovascular Outcome Data	Limitations	Refs.
Exenatide (Byetta)	Amylin/Eli Lilly (Indianapolis, ID, USA)	Twice daily SC	Immediate-release peptide	~0.8–1.0	2–3	No proven CV benefit	Injection burden; high GI intolerance; low long-term persistence	[31,34]
Lixisenatide (Adlyxin)	Sanofi (Paris, France)	Once daily SC	Modified short-acting peptide	~0.9	Modest	CV neutral (ELIXA)	Limited weight impact; modest uptake	[35,36]
Liraglutide (Victoza)	Novo Nordisk (Bagsværd, Denmark)	Once daily SC	Fatty-acid acylation (albumin binding)	~1.1–1.8	3–5	CV risk reduction (LEADER)	Daily injection; cold chain; moderate persistence	[37,38]
Albiglutide (Tanzeum/Eperzan)	GlaxoSmithKline (London, UK)	Once weekly SC	Albumin-fusion peptide	~0.8–1.6	Modest	CV benefit (HARMONY)	Withdrawn due to commercial uptake limitations	[39,40]
Dulaglutide (Trulicity)	Eli Lilly (Indianapolis, ID, USA)	Once weekly SC	Fc-fusion large molecule	~1.3–1.8	2–3	CV risk reduction (REWIND)	Improved adherence vs. daily; injection aversion persists	[41,42]
Semaglutide (Ozempic)	Novo Nordisk (Bagsværd, Denmark)	Once weekly SC	Fatty-acid enhanced albumin binding	~1.5–2.3	4–6	CV benefit (SUSTAIN-6)	High efficacy supports persistence; injection remains a barrier	[43,44,45]
Semaglutide (Rybelsus)	Novo Nordisk (Bagsværd, Denmark)	Oral (daily)	SNAC absorption enhancer	-	-	Ongoing CV/renal evaluation	Removes the injection barrier; fasting requirements; GI AEs	[46,47,48]

**Table 3:** Clinical efficacy summary of semaglutide: subcutaneous (SUSTAIN) and oral (PIONEER and Phase II) programs.

Study	Route	Duration (Weeks)	Dose (mg)	Comparator	HbA1c Reduction (%)	Weight Reduction (kg)	Clinical Context	Refs.
SUSTAIN 1	SC	30	0.5	Placebo	−1.43	−2.75	Monotherapy	[132,133,134]
			1.0	Placebo	−1.53	−3.56	Dose-dependent	
SUSTAIN 2	SC	56	0.5	Sitagliptin	−0.77	−2.35	Superior to DPP-4	[132,135]
			1.0	Sitagliptin	−1.06	−4.20	Durable efficacy	
SUSTAIN 3	SC	56	1.0	Exenatide ER	−0.62	−3.78	Weekly GLP-1 comparator	[135,136]
SUSTAIN 4	SC	30	0.5 (+insulin)	Insulin glargine	−0.38	−4.62	Weight advantage vs. insulin	[135,137]
			1.0 (+insulin)	Insulin glargine	−0.81	−6.33	High efficacy	
SUSTAIN 5	SC	30	0.5 (+insulin)	Placebo	−1.35	−2.31	Add-on insulin	[132,135]
			1.0 (+insulin)	Placebo	−1.75	−5.06	Strong metabolic effect	
SUSTAIN 6	SC	104	0.5	Placebo	−0.70	−2.90	CV outcome trial	[138,139]
			1.0	Placebo	−1.00	−4.30	Long-term durability	
SUSTAIN 7	SC	40	0.5	Dulaglutide	−0.40	−2.26	Head-to-head GLP-1	[139,140]
			1.0	Dulaglutide	−0.41	−3.55	Comparable/superior	
PIONEER 1	Oral	26	3	Placebo	−0.7	−0.2	Monotherapy	[132,141]
			7	Placebo	−1.2	−1.0	Dose response	
			14	Placebo	−1.4	−2.6	Approved oral dose	
PIONEER 2	Oral	52	14	Empagliflozin	−0.5	−0.9	Vs. SGLT2	[141,142]
PIONEER 3	Oral	78	3	Sitagliptin	0.1	−0.8	Low dose	[142]
			7	Sitagliptin	−0.3	−1.6	Superior to DPP-4	
			14	Sitagliptin	−0.7	−2.4	Durable effect	
PIONEER 4	Oral	52	14	Placebo	−1.4	−3.8	Robust metabolic effect	[143]
			14	Liraglutide 1.8 mg	−0.3	−1.9	Comparable to SC GLP-1	
PIONEER 5	Oral	26	14	Placebo	−1.0	−2.6	Moderate CKD population	[144]
PIONEER 7	Oral	52	3–14	Sitagliptin	—	−2.1	Flexible titration	[145]
Phase II	Oral	26	2.5	Placebo	−0.4	−0.9	Early proof-of-concept	[145,146]
			5	Placebo	−0.9	−1.5		
			10	Placebo	−1.2	−3.6	Comparable to SC 0.5 mg	
			20	Placebo	−1.4	−5.0	Approaching SC 1.0 mg	
			40	Placebo	−1.6	−5.7	Exposure plateau	

**Table 4:** Comparative landscape of oral vs. injectable GLP-1 RAs.

Parameter	Oral GLP-1 Receptor Agonists	Injectable GLP-1 Receptor Agonists	Critical Interpretation/Clinical Implication	Refs.
Bioavailability & Pharmacokinetics	Very low (<1%) and highly variable absorption; dependent on gastric conditions and strict dosing requirements	Near-complete systemic bioavailability; predictable and stable exposure	Injectable formulations provide more reliable pharmacokinetic profiles; oral agents introduce exposure variability that may affect real-world effectiveness	[88,96]
Dosing & Administration	Daily dosing under strict conditions (fasting, limited water, delayed food intake)	Daily or once-weekly subcutaneous administration with fewer restrictions	Oral therapy removes injection burden but introduces behavioral complexity; convenience is not absolute	[142]
Adherence & Persistence	Improved acceptance due to non-invasive route; adherence may be compromised by dosing complexity	Needle aversion may reduce initiation; once-weekly formulations improve persistence	Adherence depends on both psychological and practical factors; oral ≠ universally better adherence	[143]
Glycemic Efficacy (HbA1c Reduction)	Comparable efficacy at higher doses in controlled trials (e.g., PIONEER program)	Consistently robust and dose-dependent HbA1c reduction across agents	Trial-based equivalence may not fully translate into real-world settings due to variability in oral absorption	[132]
Weight Reduction	Clinically meaningful weight loss, dose-dependent	Greater and more consistent weight loss, especially with long-acting agents	Injectable agents maintain a slight advantage in magnitude and consistency of weight reduction	[138,139,140,147]
Cardiovascular Outcome Evidence	Demonstrated safety; long-term outcome data still emerging	Strong, well-established CV benefit across multiple large trials	Injectable GLP-1 RAs currently hold a clear evidence advantage in hard clinical endpoints	[141]
Renal Outcomes	Promising signals (albuminuria reduction, eGFR stability); long-term data limited	Established renal protective effects in multiple studies	Oral formulations require further validation to confirm equivalence in renal protection	[132]
Safety & Tolerability	Similar GI adverse effects; variability in exposure may influence tolerability	Similar GI profile; more stable exposure may improve tolerability consistency	Safety profiles are broadly comparable, but variability in oral exposure may affect patient experience	[142]
Real-World Effectiveness	Influenced by dosing adherence and physiological variability	More consistent effectiveness due to stable exposure and simpler regimens (weekly dosing)	Real-world effectiveness may favor injectables despite theoretical convenience of oral therapy	[143]
Logistics & Accessibility	No cold-chain requirement; easier storage and distribution	Requires cold-chain and injection devices	Oral formulations offer advantages in scalability and access, especially in resource-limited settings	[80]
Clinical Positioning	Suitable for early-stage disease or patients unwilling to initiate injections	Preferred in advanced disease requiring consistent and maximal therapeutic effect	Supports a stratified, patient-centered approach rather than direct substitution	[145]

**Table 5:** Competitive landscape of oral GLP-1 RAs: molecular strategy and translational outlook.

Company	Molecular Class	Receptor Profile	Development Status (2025)	Dosing Strategy	Key Difference	Renal/Cardiometabolic Positioning	Target Strategy	Refs.
Novo Nordisk–Oral Semaglutide (Rybelsus^®^/oral Wegovy^®^)	Peptide + SNAC enhancer	GLP-1 selective	Approved (T2DM); obesity indication under review	Once daily (fasting required)	First validated oral peptide GLP-1; gastric absorption platform	CV benefit established (injectable extrapolation); albuminuria reduction signals; CKD safety demonstrated	Near-term market leader; obesity expansion pivotal (2025–2026)	[148,149]
Eli Lilly–Orforglipron	Small-molecule non-peptide	GLP-1 selective (TM pocket binding)	Phase III	Once daily; food-independent	No enhancer required; scalable chemical synthesis	Strong metabolic efficacy; renal outcomes pending	Potential major market shift post-2026	[150,151]
Structure Therapeutics–GSBR-1290	Small-molecule non-peptide	GLP-1 selective	Phase II	Once daily	Biased signaling potential; oral stability	Early weight reduction; renal data not reported	Mid-term challenger dependent on durability	[152,153,154]
AstraZeneca/Eccogene–ECC5004	Small-molecule oral	GLP-1 selective	Phase I–II	Once daily	integration with AZ cardiometabolic	Strategic combination potential with SGLT2 platform	Longer development horizon	[155,156]
Roche/Carmot–CT-966	Small-molecule oral	GLP-1 selective	Early clinical	Once daily	Platform-driven metabolic expansion	Cardiometabolic positioning; renal endpoints undefined	Long-term entrant	[156]
Viking Therapeutics–VK2735 (oral)	Dual agonist small molecule	GLP-1 + GIP	Advancing clinical development	Once daily	Oral dual agonism; tirzepatide-like ambition	Potential superior metabolic efficacy; renal unknown	High-risk, high-impact candidate	[157,158,159,160]
Merck/Hansoh–HS-10535	Small-molecule oral	GLP-1 selective	Early stage	Once daily	Expands metabolic portfolio; non-peptide scaffold	Early-stage; renal data absent	Strategic diversification	[161,162]

**Table 6:** Ongoing clinical trials of oral GLP-1 receptor agonists across all phases.

Phase III								
Trial ID	Molecule	Phase	Status	Population/Indication	Study Design/Comparator	Primary Endpoint	Key Secondary Endpoints	Sponsor
NCT05869903	Orforglipron (LY3502970)	Phase 3	Active, not recruiting	Obesity/overweight with comorbidities	Placebo-controlled	% body weight change (72 wk)	HbA1c change, ≥10% weight loss	Eli Lilly
NCT06649045	Orforglipron	Phase 3	Active, not recruiting	Obesity + obstructive sleep apnea	Placebo-controlled	% body weight change (52 wk)	AHI, HbA1c	Eli Lilly
NCT06672549	Orforglipron	Phase 3	Recruiting	Pediatric obesity (12–17 yrs)	Platform trial vs. placebo	BMI z-score change	Weight, safety	Eli Lilly
NCT06672939	Orforglipron	Phase 3	Recruiting	Adolescent obesity	Placebo-controlled	% body weight change	BMI, safety	Eli Lilly
NCT05803421	Orforglipron	Phase 3	Active, not recruiting	T2D + obesity/overweight	vs. insulin glargine	HbA1c change (52 wk)	Weight change, A1c ≤ 7%	Eli Lilly
NCT06948435	Orforglipron	Phase 3	Recruiting	Hypertension + obesity	Placebo-controlled	% body weight change	BP change, safety	Eli Lilly
NCT06952530	Orforglipron	Phase 3	Recruiting	Hypertension + obesity	Placebo-controlled	% body weight change	BP change	Eli Lilly
NCT06972472	Orforglipron	Phase 3	Recruiting	T2D + obesity	Placebo-controlled	% body weight change	HbA1c, safety	Eli Lilly
NCT07153471	Orforglipron	Phase 3	Recruiting	Obesity + knee osteoarthritis	Placebo-controlled	% body weight change	WOMAC pain score	Eli Lilly
NCT07202884	Orforglipron	Phase 3	Recruiting	Women with obesity + stress urinary incontinence	Placebo-controlled	Incontinence episodes	Weight loss, safety	Eli Lilly
NCT07241390	Orforglipron	Phase 3	Recruiting	ASCVD/CKD + obesity	CV outcomes trial	MACE composite	Renal outcomes, weight	Eli Lilly
**Phase II**								
**Trial ID**	**Molecule**	**Phase**	**Status**	**Population**	**Design**	**Primary Endpoint**	**Secondary**	**Sponsor**
EUCTR 2021-002805-88	Orforglipron	Phase 2	Ongoing	Obesity + comorbidities	Placebo-controlled	% weight change (16 wk)	Safety, metabolism	Lilly Europe
EUCTR 2021-002806-29	Orforglipron	Phase 2	Ongoing	T2D	vs. placebo + dulaglutide	HbA1c change (24 wk)	Weight loss	Lilly Europe
NCT06567327	Danuglipron (PF-06882961)	Phase 2	Discontinued	T2D ± statins	Dose optimization	PK parameters	Safety, dose selection	Pfizer
**Phase I**								
**Trial ID**	**Molecule**	**Phase**	**Status**	**Population**	**Design**	**Primary Endpoint**	**Secondary**	
NCT07140055	BLX-7006	Phase 1	Recruiting	Healthy adults	Safety, PK	PD measures	Biolexis	
NCT05814107	CT-996	Phase 1	Partially completed	Overweight/obese	Safety, tolerability	Weight loss (exploratory)	Genentech/Roche	

Data compiled from ClinicalTrials.gov and EU Clinical Trials Register (EUCTR). Trial status and endpoints reflect registry entries at the time of access: March, 2026.

## Data Availability

No new data were created or analyzed in this study. Data sharing is not applicable.
